# Supplementary material for: Deficiency in cytosine DNA methylation leads to high chaperonin expression and tolerance to aminoglycosides in Vibrio cholerae
Source: PLoS Genet. 2021 Oct 20;17(10):e1009748. doi: 10.1371/journal.pgen.1009748 (PMC8559950; doi:10.1371/journal.pgen.1009748)
Supplement: S1 Table — (PDF) [file pgen.1009748.s001.pdf]

Table S1. Differentially regulated genes in *ΔvchM* strain

Locus	Name	Annotation	Fold change *ΔvchM*/WT	p-value
**UPREGULATED**				
vca1028	*lamB*	maltoporin	52,81	0,0E+00
vca0707	*uhpC*	MFS transporter	8,76	8,6E-06
vc1368		conserved hypothetical protein	8,18	0,0E+00
vca0819	***groES-2***	**chaperonin**	**6,78**	**0,0E+00**
vca0200		putative ATPase	6,10	0,0E+00
vca0957	*glcB*	putative Malate synthase	5,97	0,0E+00
vca0199		conserved hypothetical protein	5,32	5,2E-09
vca1027	*malM*	putative Maltose operon periplasmic protein MalM	4,14	0,0E+00
vca0201		conserved hypothetical protein	3,97	3,4E-11
vca0946	*malK*	Maltose/maltodextrin import ATP-binding protein malK	3,91	0,0E+00
vca0469	*higA2*	plasmid stabilization system HigA protein (antitoxin of TA system HigB-HigA)	3,91	0,0E+00
vca0468	*higB2*	plasmid stabilization system HigB protein (toxin of TA system HigB-HigA)	3,90	2,3E-10
vca0945	*malE*	maltose ABC transporter periplasmic binding protein	3,90	0,0E+00
vc1819	*aldA*	Aldehyde dehydrogenase	3,84	1,7E-12
vc1204	*hutG*	putative Formimidoylglutamase HutG (histidine utilization)	3,80	2,2E-16
vc1203	*hutU*	Urocanate hydratase (histidine utilization)	3,60	0,0E+00
vca0820	***groEL-2***	**chaperonin**	**3,54**	**2,2E-16**
vca0692	*secF*	fragment of putative SecD/SecF/SecDF export membrane protein (part 2)	3,48	3,2E-08
vc1809	*alpA*	putative Prophage CP4-57 regulatory protein (AlpA)	3,37	1,8E-09
vc1205	*hutI*	Imidazolonepropionase (histidine utilization)	3,24	1,1E-11
vc1740	*fadE*	acyl coenzyme A dehydrogenase	3,22	5,6E-15
vca0944	*malF*	maltose ABC transporter membrane subunit MalF	3,18	0,0E+00
vc1142	*cspD*	Cold shock-like protein cspD	2,88	0,0E+00
vc2530	*hpf*	putative ribosome hibernation promoting factor HPF/sigma 54 modulation protein	2,83	0,0E+00
vca0948	*yaeO*	putative Rho-specific inhibitor of transcription termination (YaeO)	2,78	0,0E+00
vca0998	*xenB*	Xenobiotic reductase B	2,73	4,4E-06

vc0486	*srlR*	putative Transcriptional regulator of sugar metabolism; DeoR family transcriptional regulator	2,65	9,9E-14
vc1874		putative SpoVR family protein	2,64	0,0E+00
vc1202	*hutH*	Histidine ammonia-lyase	2,61	0,0E+00
vc2265	*groES-1*	chaperonin	2,60	0,0E+00
vc2615		conserved hypothetical protein	2,55	4,6E-10
vc1678	*pspA*	Phage shock protein A	2,48	4,6E-10
vc1183		putative 2OG-Fe dioxygenase	2,43	0,0E+00
vca0470		putative Acetyltransferase	2,43	2,1E-08
vca0324	*relB1*	Plasmid stabilization system protein RelB (Anti Toxin of TA system RelB-RelE)	2,43	1,1E-11
vca0886	*kbl*	glycine C-acetyltransferase	2,40	2,3E-08
vca0185	*arfA*	putative Stalled ribosome alternative rescue factor ArfA	2,39	3,0E-13
vca0958		putative transcriptional regulator	2,38	3,9E-10
vca0013	*malP*	maltodextrin phosphorylase	2,37	3,9E-11
vca0881		conserved hypothetical protein	2,36	1,1E-13
vca0551		conserved hypothetical protein	2,35	0,0E+00
vca0391	*higB1*	Plasmid stabilization system HigB protein (Toxin of TA system HigB-HigA)	2,34	1,5E-10
vca0923	*mlp37*	chemoreceptor Mlp37	2,34	2,2E-12
vc2361	*grcA*	Autonomous glycyl radical cofactor	2,30	0,0E+00
vca0392	*higA1*	Plasmid stabilization system HigA protein (Antitoxin of TA system HigB-HigA)	2,28	0,0E+00
vc0665	*vpsR*	Fis family transcriptional regulator (sigma-54 dependent transcriptional regulator)	2,28	0,0E+00
vca0720	*hnoX*	Heme-Nitric Oxide/Oxygen Binding Protein (H-NOX)	2,27	5,1E-10
vc1433	*uspE*	universal stress protein UspE	2,26	0,0E+00
vc1248	*tar*	putative Methyl-accepting chemotaxis protein	2,25	0,0E+00
vc2264	*groEL-1*	chaperonin	2,24	0,0E+00
vc0734	*aceB*	Malate synthase A	2,24	1,4E-06
vc2507		PhoH family protein	2,23	0,0E+00
vc1344	*hppD*	4-hydroxyphenylpyruvate dioxygenase	2,22	6,9E-15
vca0219	*hlyA*	Hemolysin	2,20	5,7E-14
vca0159		putative Universal stress protein family 1	2,17	2,9E-15
vca0987	*ppsA*	phosphoenolpyruvate synthase	2,15	2,6E-09

vca0359	*parE2*	Plasmid stabilization system ParE protein (toxin of TA system ParD-ParE)	2,14	9,4E-10
vca0004		conserved hypothetical protein	2,14	0,0E+00
vc1222	*ihfA*	integration host factor subunit α	2,13	0,0E+00
vc2758	*fadB*	fatty acid oxidation complex subunit alpha FadB	2,10	6,1E-10
vc2704		conserved hypothetical protein	2,10	8,9E-09
vc1962	*nlpE*	copper resistance protein NlpE N-terminal domain-containing protein	2,08	3,9E-09
vca0278	*glyA*	serine hydroxymethyltransferase	2,04	0,0E+00
vca0280	*gcvT*	glycine cleavage system aminomethyltransferase	2,04	4,4E-16
vc0976	*qmcA*	putative Membrane protease subunits, stomatin/prohibitin homolog qmcA	2,03	6,5E-06
vc1539a		hypothetical protein	2,02	6,9E-10
vc0666		conserved hypothetical protein	2,02	1,8E-09
vc2144	*flaF*	Polar flagellin F	2,01	3,0E-13
**DOWNREGULATED**				
**Locus**	**Name**	**Annotation**	**Fold change Δ*vchM*/WT**	**p-value**
vca0198	*vchM*	Cytosine-specific DNA methyltransferase	-12,22	1,4E-24
vca0933	*cspE*	transcription antiterminator and regulator of RNA stability	-8,41	1,0E-07
vca0017	*hcp*	Haemolysin co-regulated protein (putative Type VI secretion system effector, Hcp1)	-3,20	1,3E-10
vc2383	*ilvY*	putative Transcriptional regulator, LysR family	-3,14	5,9E-27
vca0804	*deaD*	Cold-shock DEAD box protein A	-3,12	6,5E-49
vca0874		conserved hypothetical protein	-3,10	1,1E-05
vc1704	*metE*	5-methyltetrahydropteroyltriglutamate--homocysteine methyltransferase	-3,02	7,6E-21
vc0768	*guaA*	GMP synthetase	-2,91	2,7E-72
vca0680	*napC*	Cytochrome c-type protein napC	-2,87	2,4E-07
vca0679	*napB*	Diheme cytochrome c napB	-2,82	2,1E-06
vca0935		hypothetical protein	-2,76	1,2E-10
vc1261		putative Sugar efflux transporter B	-2,63	3,9E-07
vc0069	*mdtL*	putative FLORFENICOL EXPORTER	-2,53	8,1E-14
vca1002		AzlC family ABC transporter permease	-2,49	2,0E-08
vc1415	*hcp*	type VI secretion system secreted protein Hcp	-2,48	1,0E-06
vc0433	*arcD*	Arginine/ornithine antiporter	-2,48	1,9E-06

vc1393	*sugE*	Quaternary ammonium compound-resistance protein sugE	-2,43	3,4E-22
vc1035		conserved hypothetical protein	-2,40	6,3E-06
vc1608		ABC transporter permease	-2,38	2,8E-11
vca0250		Alpha-amylase	-2,37	7,2E-08
vc0767	*guaB*	inositol-5-monophosphate dehydrogenase	-2,31	3,0E-22
vc1239	*cobU*	cobinamide-P guanylyltransferase / cobinamide kinase	-2,28	1,2E-09
vc0290	*fis*	DNA-binding protein fis	-2,27	1,4E-04
vc2227	*purN*	phosphoribosylglycinamide formyltransferase 1	-2,25	2,6E-10
vc2384		TSUP family transporter	-2,24	8,1E-16
vca1003		AzlD domain-containing protein	-2,23	1,5E-11
vca0678	*napA*	Periplasmic nitrate reductase	-2,22	2,6E-10
vca1031		Methyl-accepting chemotaxis protein	-2,21	7,0E-11
vc1843	*cydB*	Cytochrome d ubiquinol oxidase subunit 2	-2,21	5,9E-13
vc0191	*rhtC*	putative Lysine/Homoserine/Threonine exporter protein	-2,20	2,6E-10
vc1609		ABC transporter permease	-2,19	2,2E-10
vc0005	*yidD*	membrane protein insertion efficiency factor	-2,16	4,9E-10
vc0291	*dusB*	tRNA-dihydrouridine synthase B	-2,16	3,8E-16
vc1855	*dinG*	ATP-dependent DNA helicase DinG	-2,15	7,7E-07
vc2382		conserved hypothetical protein	-2,15	3,1E-07
vc1842	*cydX*	cytochrome bd-I oxidase subunit CydX	-2,14	1,8E-06
vc1629		ABC transporter permease	-2,13	3,0E-06
vc0706	*raiA*	Ribosome-associated inhibitor A	-2,13	1,1E-08
vc2047		SDR family oxidoreductase	-2,11	3,4E-23
vc1174	*trpE*	Anthranilate synthase component 1	-2,09	9,9E-18
vc1240		histidine phosphatase family protein	-2,09	1,5E-06
vc0940		conserved hypothetical protein	-2,07	1,3E-07
vc1487		glutaredoxin family protein	-2,06	8,0E-06
vca0214	*emrD*	multidrug efflux pump EmrD	-2,05	6,0E-12
vca0269		aspartate aminotransferase family protein	-2,04	2,2E-13
vc2647	*aphA*	Transcriptional regulator PadR family	-2,04	9,6E-56
vc2226	*purM*	Phosphoribosylformylglycinamidine cyclo-ligase	-2,02	9,3E-10
vc0651		putative Peptidase U32	-2,02	2,4E-13
vc1630		putative SalX, ABC-type antimicrobial peptide transport system, ATPase component	-2,02	2,5E-19
vca0684		MFS transporter	-2,02	9,3E-14

vc0717	*yegQ*	tRNA 5-hydroxyuridine modification protein YegQ	-2,01	4,2E-09
vc1856		TSUP family transporter	-2,00	7,2E-10
vc0987	*hemH*	ferrochelatase	-2,00	1,9E-17
