# Supplementary material for: Deficiency in cytosine DNA methylation leads to high chaperonin expression and tolerance to aminoglycosides in Vibrio cholerae
Source: PLoS Genet. 2021 Oct 20;17(10):e1009748. doi: 10.1371/journal.pgen.1009748 (PMC8559950; doi:10.1371/journal.pgen.1009748)
Supplement: S2 Table — (PDF) [file pgen.1009748.s002.pdf]

Table S2. Strains, plasmids and primers used in this study

Strain	Lab Strain number	Source/Construction
Vibrio cholerae		
N16961 hapR+ WT strain	F606	Gift from Melanie Blokesch
Δ*vchM* (vca0198)	H507	PCR amplification of 500bp up and down genomic regions of VCA0198 using primers ZIP136/137 and ZIP138/139. PCR amplification of aadA7 conferring spectinomycin resistance on pAM34 using ZB47/48. PCR assembly of the VCA0198::spec fragment using ZIP136/139 and allelic exchange by natural transformation in F606.
Δ*lacZ*	K329	Lab collection
Δ*groESL-2* (*vca0819-0820*)	M958	allelic exchange by integration and excision of conjugative suicide plasmid pMP7 (pM340) replacing the gene with frt::kan::frt
Δ*vchM* Δ*groESL-2*	N330	PCR amplification of 500bp up and down genomic regions of VCA0198 using primers ZIP136/137 and ZIP138/139. PCR amplification of aadA7 conferring spectinomycin resistance on pAM34 using ZB47/48. PCR assembly of the VCA0198::spec fragment using ZIP136/139 and allelic exchange by natural transformation in M958
WT – VchM site #1 mutated (mut #1)	L900	allelic exchange by integration and excision of conjugative suicide plasmid pMP7 (pL442)
WT – VchM sites #1-4 mutated (mut #1-4)	Q827	allelic exchange by integration and excision of conjugative suicide plasmid pMP7 containing the *groESL-2* region with sites #1-4 mutated (pQ824) in M958 strain.
Δ*vchM* mut #1-4	Q828	allelic exchange by integration and excision of conjugative suicide plasmid pMP7 containing the *groESL-2* region with sites #1-4 mutated (pQ824) in N330

**Plasmids**		
pMP7-Δ*vca0819-0820*::kan	M340	gibson assembly using primers MV450/451 for the amplification of pMP7 vector, primers "vca0819-8205" and "vca0819-8206" for up and down regions of the gene, and primers MV268/269 on pKD4 plasmid for the resistance gene (frt::kan::frt).
pMP7- C->T point mutation of VchM site #1 in 5' UTR region of vca0819-0820	L442	Amplification of 500bp upstream of *vca0819* with primers 5923/5922; Amplification of 500bp downstream of *vca0819* with 5924/5921; PCR assembly of the two fragments with primers 5923/5924. Note: Primers 5922 and 5921 contain a mismatch to give origin to a C->T point mutation in VchM site #1. The fragment was then cloned in a pTOPO vector and sub cloned into pMP7 using *EcoRI* restriction sites.
pMP7- sites #1-4 mutated in vca0819-0820 region	Q824	A *groESL-2* fragment with mutations in VchM sites #2-4 was synthesized and cloned in a pTOPO vector. This fragment was then PCR assembled to the 5' UTR region containing site #1 mutated (from strain L900), which originated a final fragment containing the #1-4 mutated sites. This fragment was then cloned in pTOPO and sub cloned into pMP7 using *EcoRI* restriction sites.
pSC101-*groESL-1*	O849	Amplification of *vc2664-2665* from *V. cholerae* gDNA with primers AFC046/AFC047. Primer AFC046 contains a Ptrc promoter. Fragment cloned in pSC101 low copy plasmid (carbenicillin resistant) using *EcoRI* restriction sites
pSC101-*groESL-2*	N752	Amplification of *vca0819-0820* from *V. cholerae* gDNA with primers AFC029/AFC030. Primer AFC029 contains a Ptrc promoter. Fragment cloned in pSC101 low copy plasmid (carbenicillin resistant) using *EcoRI* restriction sites
pSC101-*vchM*	Q826	Amplification of *vca0198 (vchM)* with its own promoter from *V. cholerae* gDNA with primers 5990/5911. Cloning in pTOPO vector and sub cloning in pSC101 low copy plasmid (carbenicillin resistant) using *BamHI* and *PstI* restriction sites
**Primers**		**Sequence 5'-3'**
ZIP136		GCCGCCGAAGGAAAAACCGTACTATTGC
ZIP137		GCGAGCATCGTTTGTTCGCCCAGCTTCTGTATGGAACGGGTTAACTGTATCACCATACTACCTCATGG
ZIP138		CGTGAAAGGCGAGATCACCAAGGTAGTCGGCAAATAATGTCTACATGCTTCACAGCGTAGTCGC
ZIP139		TTAATTTCTCGAGTTTCAGATGC

ZB47		CCCGTTCCATACAGAAGCTGGGCGAACAAACGATGCTCGC
ZB48		GACATTATTTGCCGACTACCTTGGTGATCTCGCCTTTCACG
vca0819-8205		CTATTATTTAAACTCTTTCCGTTTTGCCTT
vca0819-8206		TACGTAGAATGTATCAGACTCGCCCAAGGA
5921		AAACAATCCTA**<u>T</u>**CGGCCTTTTATC
5922		GATAAAAGGCCG**<u>A</u>**TAGGATTGTTT
5923		ACTTTGATGGTACGCGCGATG
5924		GATTTATTGAGCACAACATGGCG
AFC029		GTAAGTGAATTCTTGACAATTAATCATCCGGCTCGTATAATGTGTG GAATTGTGAGCGGATAACAATTTCACACAGGAAACAGCGCCGCAT GAATATTCGTCCTTTACATG
AFC030		GTAAGTGAATTCATTACGCCGCAGACTCTTTGTC
AFC046		GTAAGTGAATTCTTGACAATTAATCATCCGGCTCGTATAATGTGTG GAATTGTGAGCGGATAACAATTTCACACAGGAAACAGCGCCGCAT GAATATTCGTCCATTACATGAC
AFC047		GTAAGTGAATTCCTGCTAAGGGGGATGATTACA
5990		GTTTTTGCTGCCGTCTGCTA
5911		GTAGTCGACCCTTTTTACAAACTTTCTAGA
