# Supplementary material for: Deficiency in cytosine DNA methylation leads to high chaperonin expression and tolerance to aminoglycosides in Vibrio cholerae
Source: PLoS Genet. 2021 Oct 20;17(10):e1009748. doi: 10.1371/journal.pgen.1009748 (PMC8559950; doi:10.1371/journal.pgen.1009748)
Supplement: S3 Table — (PDF) [file pgen.1009748.s003.pdf]

Table S3. Primer and probe sequences used in digital qRT-PCR

Target	Primers (5’-3’)	Probes (5’-3’)
gyrA	AATGTGCTGGGCAACGACTG	[Cy5]-CACCCTCATGGTGACAGTGCGGTTT-[BHQ2]
	GAGCCAAAGTTACCTTGGCC	
groES-1	CGTAGCTTTCTGCGAAGATC	[HEX] -AGCTCCAAAGGTTGAACTGAACCGTTCTCTA-[BHQ1]
	TGGTGGAATTGTTCTAACTG	
groES-2	GGCGACCAGATCATTTTCAAC	[FAM] - TGGACGGTAAAGAGTATCTGATCCTCTCC-[BHQ1]
	TCTACAATCGCTAACACATCAG	
groEL-2	TGCTATCGTTCAGAATCAACC	[HEX] –AAGCTTACCAGCATAGAACTTGCCAGAGAT-[BHQ1]
	TTTACTTCTCGCTCCCAATCG	
