# Supplementary figures and images for: Deficiency in cytosine DNA methylation leads to high chaperonin expression and tolerance to aminoglycosides in Vibrio cholerae
Source: PLoS Genet. 2021 Oct 20;17(10):e1009748. doi: 10.1371/journal.pgen.1009748 (PMC8559950; doi:10.1371/journal.pgen.1009748)

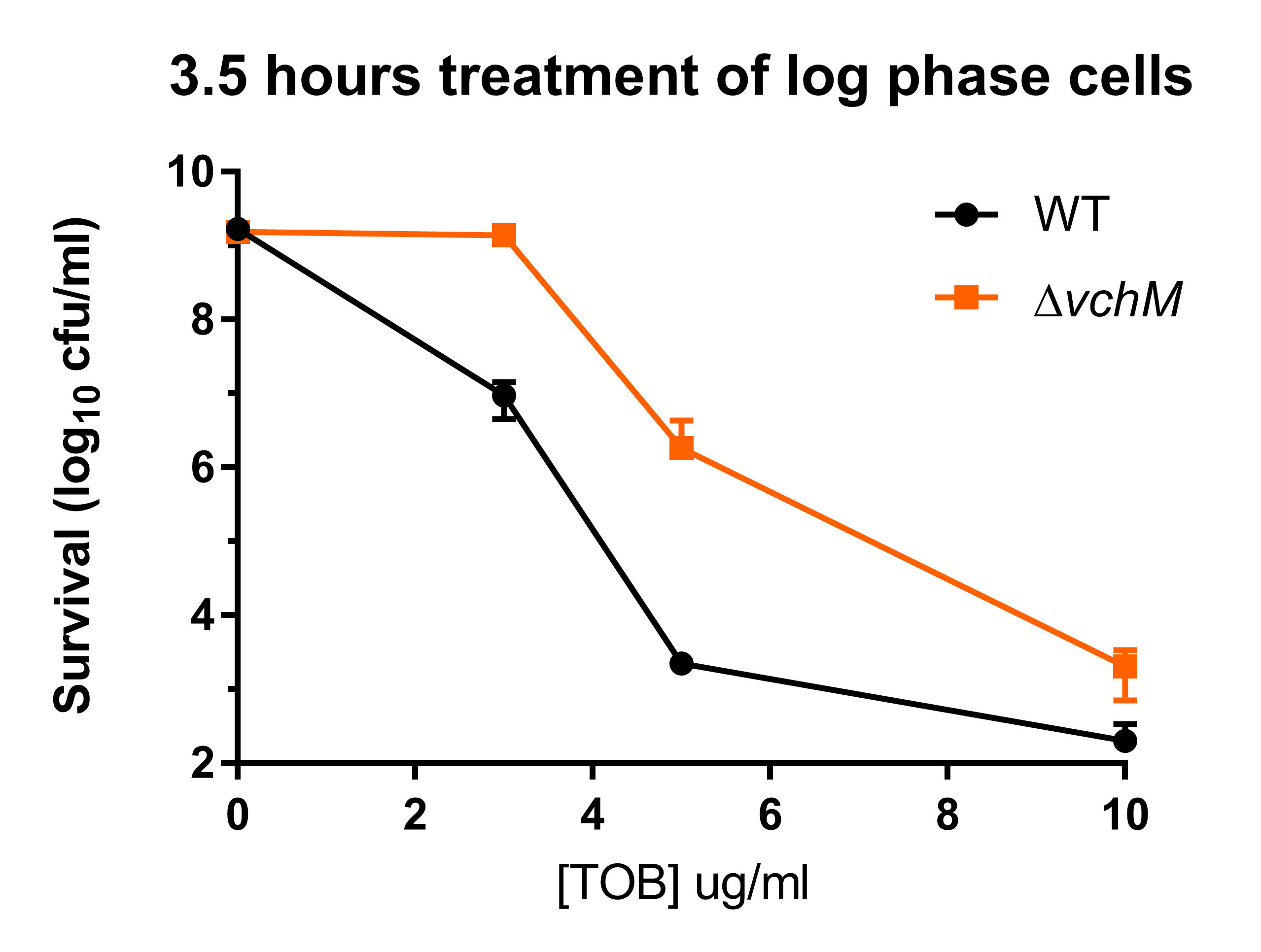

Supplement: S1 Fig — Survival of exponential phase cells (OD600 0.3–0.4) of WT and ΔvchM strains exposed to lethal doses of tobramycin (TOB) for a period of 3.5 hours. The Y-axis represents the log10 CFUs/mL after 3.5 hours growth with 0, 3, 5 or 10 μg/mL of Tobramycin (3x, 5x or 10x higher than the MIC, respectively). Means and SD are represented, n = 3. (TIF) [file pgen.1009748.s004.tif]

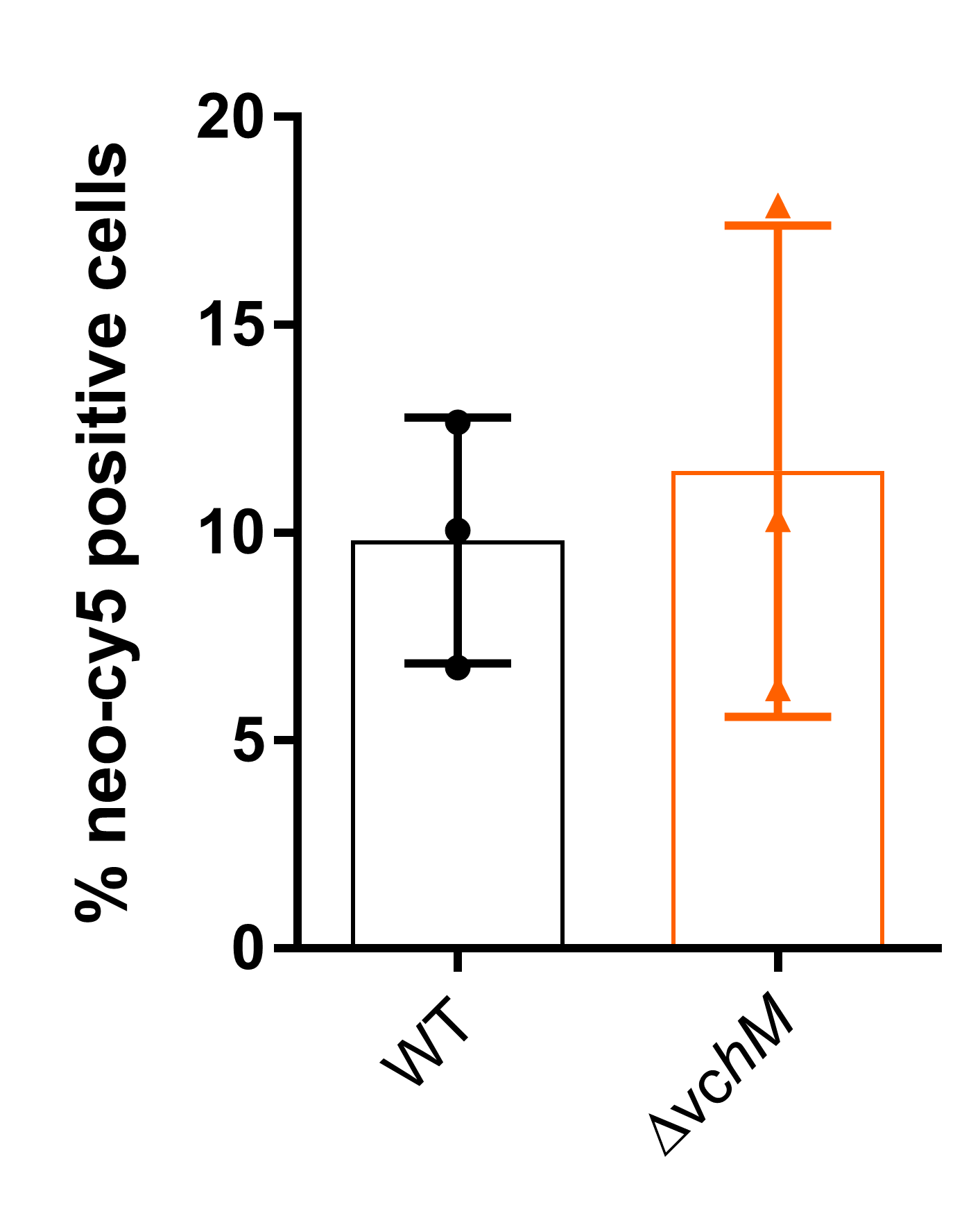

Supplement: S2 Fig — Percentage of neo-cy5 positive cells analyzed by flow cytometry after incubation with fluorescent marked neomycin. Means and SD are represented, n = 3. (TIF) [file pgen.1009748.s005.tif]

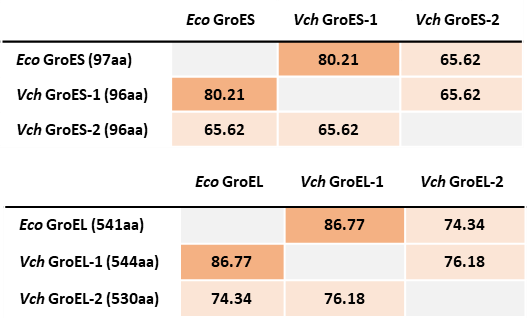

Supplement: S3 Fig — Amino acid identity between GroES and GroEL proteins of E. coli MG1655 (Eco) and V. cholerae O1 El Tor N16961 (Vch) computed by BLASTP. Values represent percentage identity between proteins. (TIF) [file pgen.1009748.s006.tif]

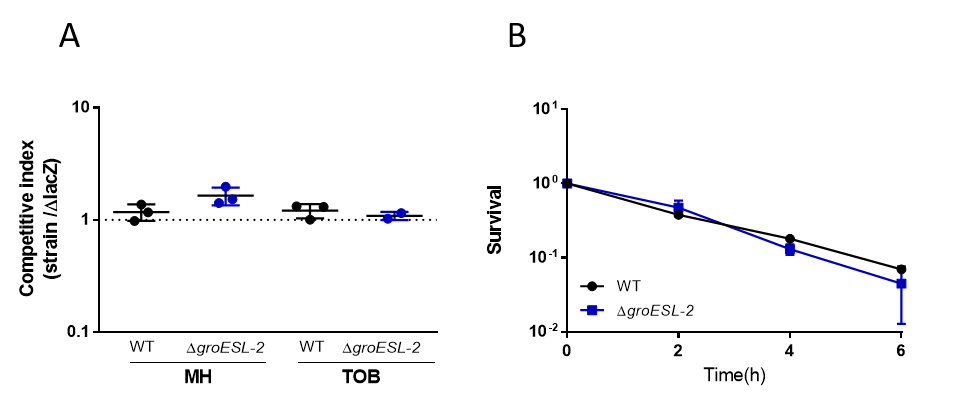

Supplement: S4 Fig — A. In vitro competitions of WT and ΔgroESL-2 strains against isogenic ΔlacZ reference strain in absence or presence of tobramycin (TOB) at 0.6 μg/mL; n = 3, error bars indicate SD. B. Survival of stationary-phase WT and ΔgroESL-2 cells exposed to 20X MIC of tobramycin. n = 3, error bars indicate SD. (TIF) [file pgen.1009748.s007.tif]

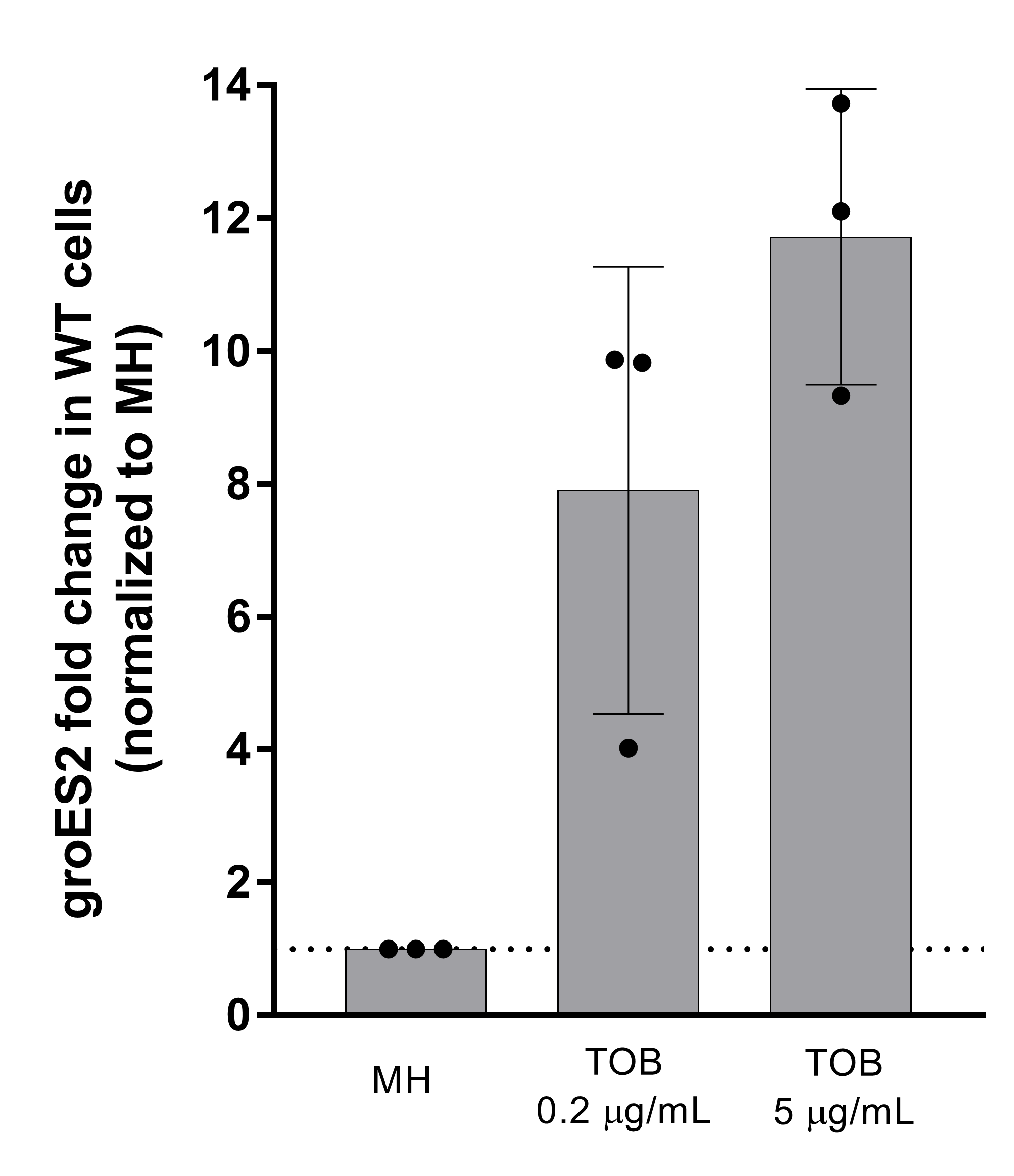

Supplement: S5 Fig — Relative expression of groES-2 in WT cells growing in MH, MH with subMIC tobramycin (≈ 20% MIC) or exponential phase cells following lethal TOB treatment (5X MIC) for a period of 30 minutes. n = 3, error bars indicate SD. (TIF) [file pgen.1009748.s008.tif]

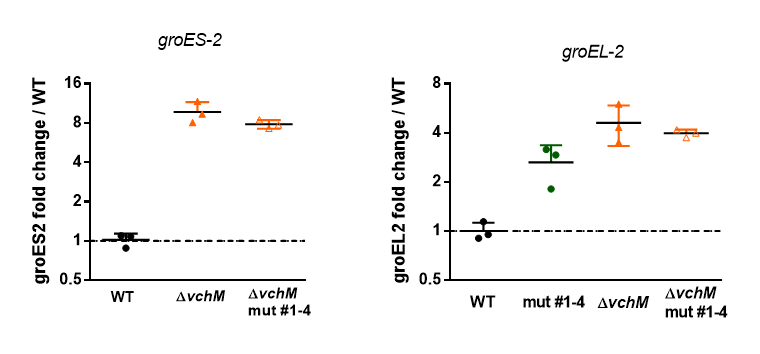

Supplement: S6 Fig — Relative expression of groES-2 and groEL-2 genes in the indicated strains grown at OD600 1.0. n = 3, error bars indicate SD. (TIF) [file pgen.1009748.s009.tif]

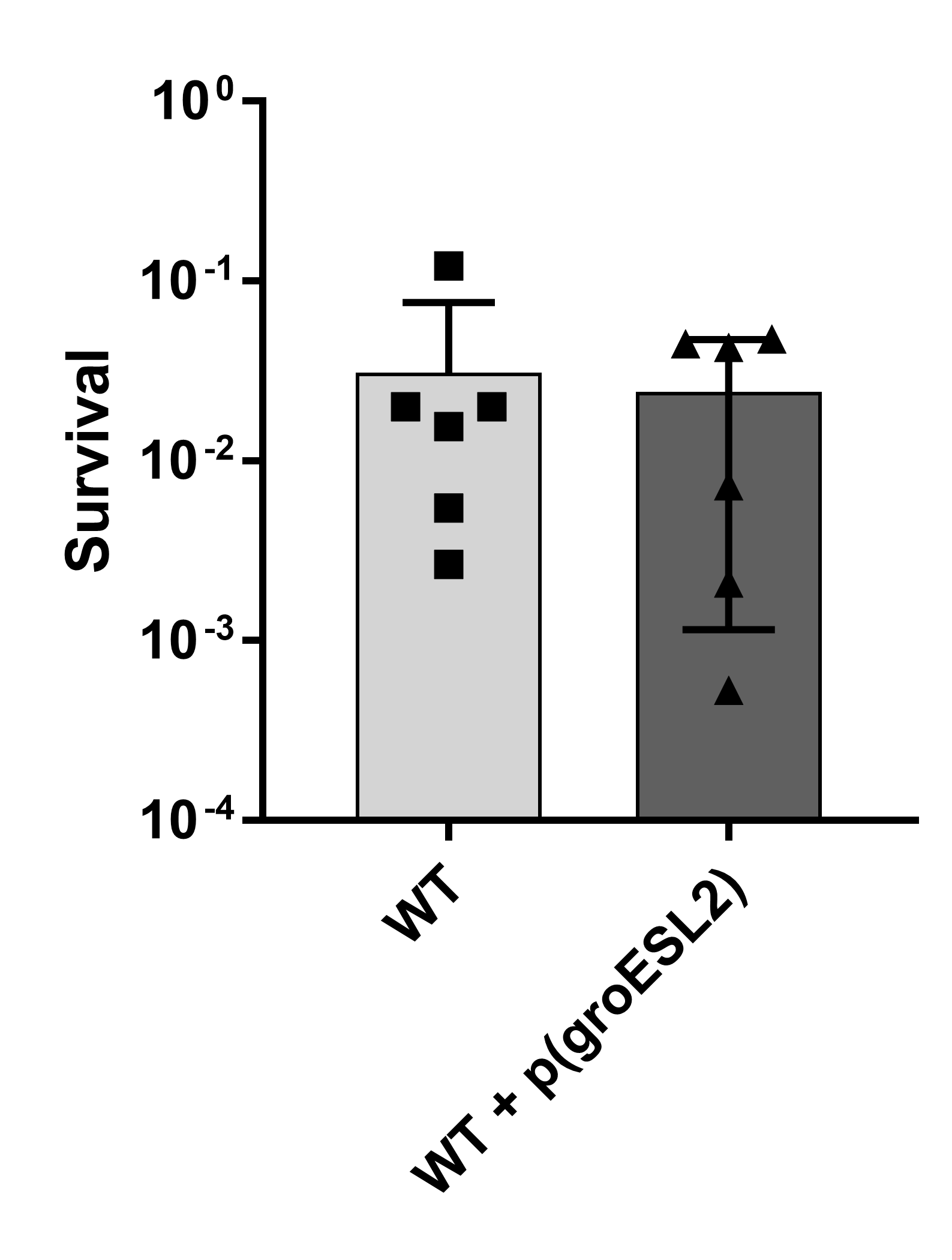

Supplement: S7 Fig — Survival (after 7 hours TOB treatment) of WT strain carrying a control plasmid or a plasmid overexpressing groESL-2 genes. n = 6, error bars indicate SD. (TIF) [file pgen.1009748.s010.tif]
